# Strategy to Identify Infants with Hypoxic Ischemic Encephalopathy for Therapeutic Hypothermia—A Retrospective Audit

**DOI:** 10.3390/children12070892

**Published:** 2025-07-07

**Authors:** Kristen Haakons, Kaycee Hocking, Richard Mausling, Helen G. Liley

**Affiliations:** 1 Mater Research, Faculty of Health, Medicine and Behavioural Sciences, The University of Queensland, Brisbane, QLD 4101, Australia; kristen.haakons@mater.uq.edu.au; 2 Newborn Medicine, Mater Mothers’ Hospital, South Brisbane, QLD 4101, Australia; kaycee.hocking@mater.org.au (K.H.); richard.mausling@mater.org.au (R.M.)

**Keywords:** neonate, hypoxic ischemic encephalopathy, audit, clinical pathway, decision aid

## Abstract

Background/Objectives: Although there is a critical need for timely, accurate recognition of infants with hypoxic ischemic encephalopathy (HIE) eligible for therapeutic hypothermia (TH), there is little published literature that comprehensively validates strategies to achieve this. For the Mater Mothers’ Hospital, a screening protocol combining use of umbilical cord gases according to obstetric criteria and other evidence of depression at birth with a decision aid (the HIE Trigger Tool (TT)) for at-risk infants was developed. We audited whether the protocol supported appropriate clinical decisions. Methods: Obstetric records were searched from 1 January 2016 to 31 July 2022 for eligible infants. Neonatal records were examined to assess usage, determine outcomes (diagnosis of HIE or other neurological conditions, use of TH, mortality and neurodevelopmental outcomes) and detect any additional HIE cases. Results: Of 64,055 live births ≥35 weeks, 35.4% had cord gases taken. Of 580 eligible infants, the TT was applied to 498 (86.3%), 155 of whom screened positive for HIE (any severity). Of 76 infants with moderate or severe encephalopathy, 69 received TH. The other seven had contraindications to TH (*n* = 2), late presentations without any depression at birth (>6 h, *n* = 3) or other causes of their encephalopathy (*n* = 2). The TT (which per instructions was commenced by one hour of age) was used to identify 61 of the infants with moderate/severe encephalopathy, while 15 were diagnosed before it was applied. No infants who screened negative using the TT presented later with seizures or any other signs of moderate or severe HIE. Conclusions: The protocol including cord gases and the HIE TT is an effective method of screening for acute HIE needing TH.

## 1. Introduction

Neonatal hypoxic ischemic encephalopathy (HIE) occurs after a period of decreased blood flow and oxygenation to the brain but may occur with or without a history of a recognizable obstetric “sentinel event” and with or without a history of advanced resuscitation, increasing the difficulty of identifying at-risk infants [1]. HIE remains an important cause of neonatal mortality and long-term disability. In countries with high healthcare resources, the incidence has been estimated at 1–5 per 1000 live births [1,2,3]. Survival and neurodevelopmental outcomes depend on the severity of HIE, which is most commonly assessed using modifications of a scale originally developed by Sarnat and Sarnat [4,5]. Infants with moderate or severe encephalopathy are at high risk of death or serious long-term disability [6].

Together with supportive intensive care, the initiation of therapeutic hypothermia (TH) within 6 h of injury can reduce death or disability in infants with moderate or severe HIE [6]. Benefits include reduced incidence of neurodevelopmental disability, developmental delay, blindness and short- and long-term mortality [6,7,8]. However, TH can distress families. To tolerate it, many infants require sedative or analgesic drugs. In addition, it can temporarily impair organ function, alter pharmacokinetics and interfere with physical contact with parents and establishment of breastfeeding, among other complications [9,10]. Therefore, it is important to find the balance between infants who have potential to benefit from TH but also avoid using it in infants for whom the burdens and risks may outweigh the benefits. The decision to initiate TH should depend on available evidence of burdens and benefits, with awareness of parent perspectives, gaps in knowledge, considerations for individual infants (including those who may have been under-represented in clinical trials) as well as resource implications.

At present, there is no gold standard test for diagnosis of moderate or severe HIE against which test characteristics of a new detection strategy, such as sensitivity and specificity, can be compared. There are international differences in criteria for initiating TH [11] and valuable recent attempts to achieve consensus for diagnosing HIE [12]. A review of guidelines from various hospitals and states in Australia revealed that most utilize eligibility criteria resembling those for the original TH trials [6] that include evidence of depression at birth together with subsequent clinical signs of encephalopathy [4,5]. Regardless of the exact criteria used, there is a need for pragmatic strategies that prospectively identify infants at risk and screen them for signs of encephalopathy within the 6 h time window when TH is considered most likely to be effective. In the absence of any gold standard test or method of detection, there is a need for an audit of clinical protocols to determine whether cases of moderate or severe HIE are being missed. However, in most reports to date that describe results of screening strategies, the potential for missed cases is not fully evaluated (Supplementary Section S1) [13,14,15,16,17,18,19,20,21,22,23]. We note that the specific aims of these studies varied, and many were not specifically intending to comprehensively audit the effectiveness of a screening strategy, so these omissions do not detract from the other value of the articles. Ideally, any detection strategy should also avoid over-treatment, but over-treatment is harder to define because of clinical uncertainty as to whether TH should be used in some cases of mild HIE. Some studies have identified a shift in practice to include many such infants [24,25], but the benefit remains uncertain, and most Australian state and hospital guidelines collected by the authors at the time of writing continue to stress the identification of moderate or severe HIE as a criterion for TH.

To screen for eligibility for TH, our hospital devised a two-step clinical strategy using cord blood gases according to a set of criteria (Supplementary Section S2) plus a decision aid, titled the “HIE Trigger Tool” (TT). The TT (Supplementary Section S3) was based on entry criteria for previous clinical trials [26] and was introduced in June 2015. The first step reminds doctors, nurses and midwives to identify infants ≥ 35 weeks’ gestation at birth who meet one or more criteria for depression at birth (Apgar ≤ 5 at ten minutes, or cord or neonatal gas with a pH <7.0 or base excess worse than −12 mmol/L, or the need for ongoing resuscitation at ten minutes). The infant is then admitted and assessed hourly by nursing or medical staff for clinical signs of encephalopathy using a simplified Sarnat scoring system [4,26], for the first six hours of life. The nursing or medical staff were, in the first instance, neonatal nurses or nurse practitioners or medical staff in training positions (e.g., registrars) who have training and familiarity with physical examination and assessment of newborn infants. When any infant meets the “moderate” or “severe” criteria in any three components in a given hour, or if they meet the criteria in two components but develop seizures, they are discussed with a neonatologist for a decision about initiating TH. Some NICUs have 24 h availability of sufficient equipment and skilled personnel to use aEEG as a screening tool for diagnosing moderate or severe HIE. Our resources are more limited, and so aEEG is used more selectively, including as part of a more extensive evaluation (including full neurological examination) in infants where the TT results are equivocal, for continuous monitoring of infants with HIE who are determined to need TH or those require monitoring for encephalopathy or seizures due to other causes.

The aim of this study was to perform a retrospective audit on the use of these combined strategies to assess whether they correctly identified all infants currently eligible for TH. The audit also allowed an informal assessment of the workload imposed on the neonatal unit by using TT. In addition, we aimed to provide a comprehensive set of data (including the denominator of liveborn infants, number eligible for screening, number screened, outcomes of screened infants and potential missed cases) by which other centers may be able to benchmark their own screening protocols

## 2. Materials and Methods

This retrospective single-center cohort study was conducted as a clinical audit. The study assessed the detection of infants eligible for TH by a combined approach using a policy for obtaining cord blood gases (Supplementary Section S2) and a policy for applying a decision aid, the HIE TT (Supplementary Section S3). We used routinely collected data available in scanned or electronic medical records at the Mater Mothers’ Hospital, a large urban teaching hospital in Brisbane, Australia, for births between 1 January 2016 and 31 July 2022. The study was approved as low or negligible risk with a waiver of the need for parental consent by the Mater Misericordiae Ltd. Human Research Ethics Committee (HREC/MML/54765).

### 2.1. Recognition of Infants Who Were Depressed at Birth

We searched data that are routinely collected in Mater Hospital’s maternity database for infants who met the following inclusion criteria: ≥35 + 0 weeks gestation and any one of cord blood pH <7.00, cord blood base excess worse than −12 mmol/L or Apgar score at 10 min ≤5. Two additional criteria for initiating the TT, “continued need for resuscitation at 10 min” and “neonatal blood gas with pH <7.00 or base excess worse than −12 mmol/L” are not recorded reliably in any field in the maternity database and therefore were not searched. (Among infants found in the neonatal database who did receive TT screening, these were very infrequent reasons for activating screening.)

To detect all cases of HIE, records of all ≥35-week gestation infants admitted to the NICU during the study interval were screened in the neonatal database. The search terms included any reference to use of the TT or any clinical diagnoses and treatments used in any inborn infant that suggested or confirmed a diagnosis of neonatal encephalopathy, HIE or seizures. Clinical records of infants identified in this search were then reviewed to confirm eligibility and whether a diagnosis of HIE had been applied. The resulting file was combined with that of infants identified through the maternity database, and duplicate cases were excluded. Infants who had a diagnosis of HIE but had not had the TT applied were categorized as “missed” opportunities for recognition, unless a diagnosis of HIE had been made on clinical grounds before the TT had been commenced.

### 2.2. Exclusion of Ineligible Infants and Determination of Whether the TT Was Applied

Infants who were stillborn or died before admission to the NICU, infants with an antenatal plan for palliation and infants who had a major congenital anomaly that would preclude consideration of TH or confound the diagnosis of HIE were excluded.

### 2.3. Audit of Completion of the HIE TT

A review of scanned patient records was used to determine whether the TT was correctly completed for each eligible infant and whether it could be interpreted to assign a level of encephalopathy. Other data items collected included whether TH was commenced, magnetic resonance imaging (MRI) results and clinical outcomes to discharge and last available follow-up. Infants admitted for TT screening who were found to have no or mild encephalopathy were also tabulated. Results from developmental follow-up were also retrieved from the neonatal database. We included any form of motor impairment, cerebral palsy, sensory issues such as hearing or visual impairment or global developmental delay detected using standardized, age-appropriate tests and multidisciplinary assessment (pediatrician, physiotherapy, developmental psychologist) where the information was available.

### 2.4. Assessment of Workload

This consisted of evaluation of the numbers of infants admitted for application of the HIE TT and the yield in terms of the proportion of infants who went on to receive TH. The workload resulting from the protocol for umbilical cord gases was not assessed, because it pre-dated the introduction of the HIE TT.

### 2.5. Data Management and Statistics

Case records for any infants for whom the diagnosis of moderate or severe HIE was considered equivocal or who were diagnosed with moderate or severe HIE but did not receive TH were reviewed by at least two of the authors to achieve consensus about the diagnosis and to assess whether the case represented a missed opportunity for use of the TT or for application of TH. Data were managed and descriptive statistics were calculated in Excel (Microsoft^® ^Redmond, WA, USA).

## 3. Results

### 3.1. Characteristics of Audited Infants

**Figure 1:**
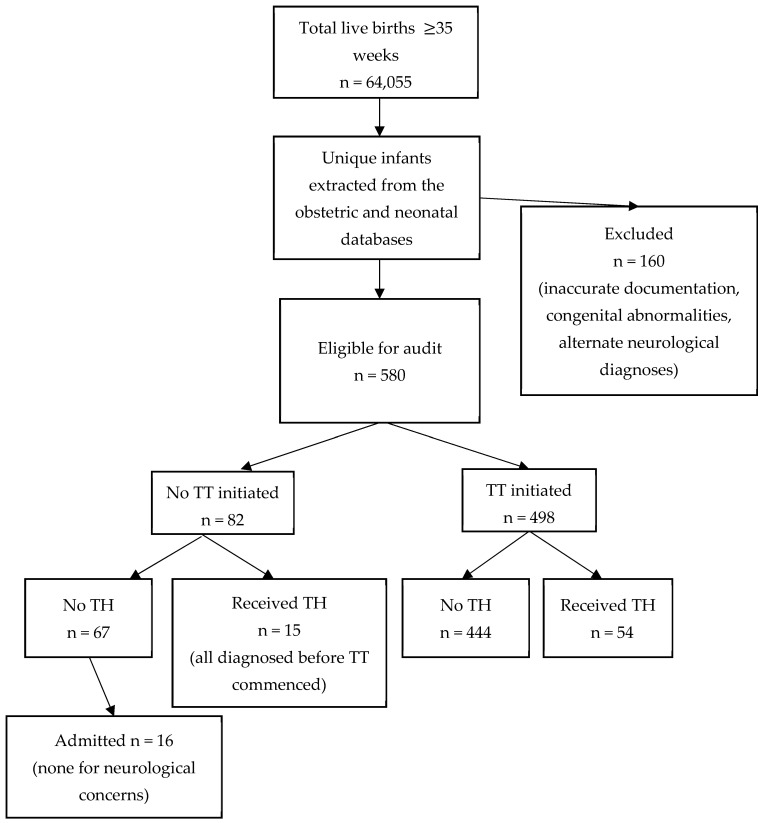
Infants identified from the obstetric and neonatal databases. Abbreviations: TH = therapeutic hypothermia; TT = Trigger Tool.

Of 64,055 live infants ≥35 weeks’ gestation born during the study interval, 22,702 (35.4%) had cord gases taken. A total of 740 infants were identified through the maternity and neonatal databases (after elimination of duplicates) as meeting cord gas thresholds or the other criteria for depression at birth. Median gestation was 39 + 1 weeks (range 35–43 weeks), median birth weight was 3.3 kg (range 1.76–5 kg) and 388 (52.4%) were male. Among them, 160 infants met one or more exclusion criteria resulting in 580 infants eligible for the audit (9.1 per 1000 live births ≥35 weeks’ gestation). The TT was initiated for 498 (85.8%). Of the eligible infants who did not have a TT initiated, 15 (18.3%) had TH commenced based on clinical assessment of moderate to severe encephalopathy before initiation of the TT (Figure 1), leaving 67 (81.7%) for whom there was a missed opportunity to apply the TT.

### 3.2. Audit of Completion of the TT

On audit of the 498 TT assessments, errors were found in those for infants who both did and did not receive TH. Two hundred and sixty-one (52.5%) were completed correctly as intended by the protocol. Most errors were either a failure to record the exact time of each examination or ambiguous labeling of the degree of severity for score components.

Among the infants who had incorrectly completed TT assessments but were not selected to receive TH, two were scored on the TT form as having moderate encephalopathy in the first one to two hours but did not receive TH. However, neither had any subsequent neurological diagnosis during the current or any later NICU admission that was consistent with a diagnosis of moderate or severe HIE. Medical record review identified that they were cases of over-detection using the TT where subsequent examination allayed concerns for moderate or severe HIE. Of the 199 infants who had errors in their TT completion but were not scored as moderate or severe HIE, none had any subsequent clinical concerns for HIE.

### 3.3. Incidence of HIE

Of 155 infants diagnosed with any stage of HIE at any time during hospital admission (incidence; 2.4 per 1000 live births ≥35 + 0 weeks plus days gestation), 72 (46.5%) were diagnosed with mild, 55 (35.5%) with moderate and 21 (13.5%) with severe HIE. For seven infants (4.5%), the conclusion about the severity of HIE was unclear from the clinical notes. The TT was used to identify 60 (79%) of the infants who were diagnosed with moderate and severe HIE. Of the 155 infants with any degree of HIE, 69 (44.5%) received TH for an incidence of 1.08 per 1000 live births (Table 1). No infant in whom the TT identified mild HIE received TH. All cooled infants received TH for 72 h except for two infants who died before completing treatment.

### 3.4. Outcomes Among Infants with Missed Opportunities to Utilize the TT for Screening

Of the 67 eligible infants who met criteria for depression at birth but did not have the TT applied and were not treated with TH, only 16 (23.9%) were admitted to the NICU at any stage (Figure 1), mostly for conditions such as hypoglycemia or respiratory distress. None had any neurological abnormalities recorded during their NICU admission.

### 3.5. Were There Any Missed Opportunities for Provision of TH?

Among the seven infants who were recorded in the neonatal database as having HIE who did not receive TH, a review of case records indicated that two infants had contraindications to TH. For two infants, other etiologies of encephalopathy were assigned (in one, abstinence from maternal medications, and in another, hypoglycemic encephalopathy). Three infants who did not receive TH were only diagnosed with possible HIE retrospectively (after MRI scanning performed for onset of neurologic abnormalities with first onset after 6 h of age), but none of these infants had met any criterion for use of the TT at birth (Table 1). Each had a late presentation, atypical for HIE. These cases may represent antenatal or postnatal asphyxia events that did not cause acidosis or depression at birth, potentially exacerbated by other contributors to cerebral energy failure (in one case, fetal anemia, in another, hypoglycemia and in the third, mild respiratory failure requiring continuous positive airway pressure). It is unclear whether therapeutic hypothermia would have benefitted these infants.

### 3.6. What Were the Outcomes of Infants in This Audit Who Received TH?

All infants who received TH were eligible for comprehensive medical and neurodevelopmental follow-up until at least 2 years of age. At the age of the last follow-up, 15 infants had died (21.7%), and 17 infants (24.6%) were found to have neurodevelopmental disability. Thus, 46.3% had either death or disability among those for whom outcome data were available. Of note, nine infants (13%) of the 69 who were cooled were lost to follow-up, and seven infants (10.1%) were less than 12 months of age at the last follow-up.

## 4. Discussion

In this retrospective cohort study, we found that all infants who were eligible for TH were recognized and received TH, except for the two for whom a clinical decision was made at the time that they had contraindications to it. Three additional possible cases of HIE did not present with any criteria for depression of birth and were found to have neurological signs only after 6 h of age, so despite suggestive MRI results, they may have had other reasons for cerebral energy failure causing the MRI abnormalities or may have had antenatal or postnatal hypoxia that did not cause depression or acidosis at birth.

Potential limitations of the study included that most, but not all infants who were eligible for assessment using the TT protocol were admitted to the NICU and evaluated. However, importantly, none of the infants who screened negative went on to be admitted to the NICU for neurological concerns. Our study period began within a few months of initiation of the TT, which may have led to a lack of familiarity with the tool early in the study. Perhaps because of this, we found that nearly half of the performed TTs had one or more errors in completion; however, this did not appear to lead to misclassification of any infants who subsequently manifested moderate or severe HIE. This suggests that once established in practice, the TT is feasible for neonatal nurses and medical officers to complete and generally leads to appropriate consideration for TH. The audit did not find any cases of moderate or severe HIE where the criteria for initiating the TT were met and the opportunity to use TH was missed. The completion errors could cause bias, but we found only two cases of possible over-detection (infants not considered to have moderate or severe HIE on more detailed examination). We would be more concerned if we had found evidence that we were missing cases of HIE, whereas our audit of neonatal records did not identify any definite missed cases among infants for whom the form had been correctly or incorrectly completed.

In assessing whether our study was likely to have identified infants appropriate for TH, we note that our overall incidence of HIE in this cohort of live births ≥35 weeks’ gestation was consistent with other published rates of HIE [1,2,3]. Furthermore, our ratio of moderate to severe HIE (2.6:1) and the combined incidence of mortality and neurodevelopmental impairment (46.3%) among the infants undergoing TH for moderate or severe HIE are not unexpected when compared to the Cochrane systematic review on TH (46.0%), suggesting that appropriate infants were selected for TH [6]. The overall mortality rate of HIE in our cohort was 12.3%, which was about half that found in the original randomized controlled trials of TH [6] and similar to the 10.2% for moderate or severe HIE in a recent, large single hospital audit [17]. This could reflect a general improvement in clinical care, the effectiveness of recognizing the need for TH early, or greater optimism about outcomes (and fewer decisions to withdraw intensive care) in an era when use of TH and subsequent early neurodevelopmental intervention have become standard. In the absence of a gold standard test for moderate or severe HIE, this comparison together with the lack of missed cases is the best evidence we can provide to support a conclusion that an appropriate group of infants was identified.

The overall success of our approach undoubtedly depends on our hospital’s protocol for obtaining umbilical cord gases in at-risk cases, and results will not be replicated using the TT alone without such a protocol (Supplementary Section S2). Some hospitals use universal cord blood gas analysis [17], but we suggest that at the least, a protocol similar to ours is warranted. In this setting, the TT was used to identify 79% of the infants who were diagnosed with moderate and severe HIE. These findings suggest that in a population with a standard incidence of HIE, and in the setting of a protocol for measuring umbilical cord gases for a range of obstetric indications, our TT is an effective clinical decision aid for accurate and timely recognition of infants who require TH.

The aim of our strategy was to create a feasible and standardized method of screening infants at high risk of HIE without dramatically increasing the clinical workload of an already busy NICU. The number of infants who received TT screening during our study period was equivalent to one to two per week in a birth service with over 10,000 births per year. For every eight infants eligible for screening, one required TH. These results suggest that applying the HIE TT is feasible and does not result in excessive admission of at-risk infants, and we speculate that it is likely to be cost-effective when evaluated against the risk of missed cases. While much current research in HIE is examining novel biochemical or electroencephalographic markers of severity, the appeal of our approach is its accessibility and ease of use, which is likely to apply even outside of a tertiary care facility. Although this retrospective study was performed on inborn infants in a tertiary NICU, we have demonstrated that in the absence of advanced laboratory or clinical tools, clinicians of varying degrees of experience can recognize infants who are potential beneficiaries of TH by using the TT protocol.

The strengths of this study are complete ascertainment of cases from a hospital with a large birth population and a tertiary NICU. This is also one of the only published studies to look for evidence of missed opportunities for TH provision as part of the auditing process [13,15,16,17,18,19,20,21,22,23]. Limitations include that only three of the five criteria for use of the TT could be readily detected in the maternity database, although we did search the neonatal database for additional cases where the TT had been used, meaning that the ascertainment of infants screened using the TT is likely to be complete. The neonatal records indicated that the other two criteria (prolonged resuscitation and neonatal rather than umbilical cord blood gases) were uncommon reasons to commence the TT, but we acknowledge that there could be some underestimation of the number of infants who were eligible for use of the TT. As noted in the results, the review of case records of the three infants with possible late onset of HIE signs found that none of the three met any criterion, including prolonged resuscitation or abnormal neonatal blood gases, for application of the TT, so we do not think they comprise missed opportunities to screen using our current criteria. We also do not have a comparison period before introduction of the TT strategy because of a major change in hospital digital records that precluded accurate comparison with any earlier interval. However, the point of the study was not to demonstrate an improvement in detection of HIE in one hospital but to provide a benchmark for our own performance for future audits and against which other strategies and services can be compared.

## 5. Conclusions

We conclude that our strategy, combining protocols for umbilical cord gases and use of the HIE TT, is an effective approach to identify infants with moderate to severe HIE who may benefit from TH. The overall strategy offers low-cost, low-burden screening that can be utilized in a variety of clinical settings by clinicians with relatively limited training beyond the ability to recognize risk factors and perform a standard neonatal exam. The success of the TT appeared resilient to errors in completion, and its use resulted in what the authors considered was an acceptably low number of additional NICU admissions for screening in proportion to the detection rate for this serious but potentially treatable condition.

## Figures and Tables

**Table 1:** Summary of the performance of the TT in identifying possible candidates for TH.

	Number	Comments
Birth population (liveborn ≥ 35 weeks)	64,055	
Met TT criteria for depression at birth	740	
Ineligible * for TT	159	
Eligible for TT	580	
Screened using TT	498	83.3% of eligible
Infants with moderate or severe HIE who received TH • detected using TT	54	All 69 received TH
• diagnosed before commencing TT	15	
Infants with encephalopathy who did not receive TH	7	HIE but contraindications to TH (*n* = 2) Other encephalopathy (not HIE) (*n* = 2) Possible late diagnoses of HIE (*n* = 3)
Potential missed opportunities to screen (using TT criteria)	67	None had any neurological diagnoses or treatments during hospital stay
Potential missed opportunities for TH (screened positive for moderate/severe HIE with TT, but did not receive TH and did not have contraindications to TH)	0 **	

* Excluded as ineligible: Infants who were stillborn or died before admission to the NICU, infants with an antenatal plan for palliation and infants who had a major congenital anomaly (e.g., congenital heart disease, chromosomal abnormality). ** One infant had mild HIE identified by TT and did not receive TH, subsequent abnormal MRI, global developmental delay. Abbreviations: TT, Trigger Tool; TH, therapeutic hypothermia; HIE, hypoxic ischemic encephalopathy; MRI, magnetic resonance imaging.

## Data Availability

The data presented in this study are available on request from the corresponding author, subject to approval of an application specifying the use to the Mater Misericordiae Ltd. Human Research Ethics Committee (HREC/MML/54765).

## Supplementary Materials

The following supporting information can be downloaded at: https://www.mdpi.com/article/10.3390/children12070892/s1, Supplementary Section S1. Summary of previous published work evaluating screening or HIE management processes compared with the current study. Supplementary Section S2. List of criteria for cord blood gases at the Mater Mothers’ Hospital. Supplementary Section S3. Mater Mothers Hospital HIE Trigger Tool.

## Abbreviations

The following abbreviations are used in this manuscript:

HIE	hypoxic ischemic encephalopathy
TH	therapeutic hypothermia
TT	Trigger Tool
NICU	neonatal intensive care unit
