# Supplementary material for: Strategy to Identify Infants with Hypoxic Ischemic Encephalopathy for Therapeutic Hypothermia—A Retrospective Audit
Source: Children (Basel). 2025 Jul 7;12(7):892. doi: 10.3390/children12070892 (PMC12294090; doi:10.3390/children12070892)
Supplement: Supplementary file 1 [file children-12-00892-s001.zip › children-3706039-supplementary.pdf]

Supplementary Section S1

Summary of previous published work evaluating screening or HIE management processes compared with the current study.

**Lead Author Year of Publication Country Location Type of study;**	**Inclusions; Inclusion dates; Eligible birth population; Stated aim of study**	**Step 1 Initial eligibility for screening** # screened positive (% of live birth population screened)	**Subsequent screen for mod/sev HIE;** # screened	**Obstetric criteria for cord gases defined?** n= (% of eligible birth population) who had cord gases)	**Cases detected** (% of birth pop.) n=missed (% of screened)	**Other results and comments**
***(Current study)* Haakons 2025 Australia** Mater Mothers' Hospital Retrospective cohort study	Inborn ≥35 wks; Jan 2016 to July 2022; N=64,055 live births *Does hospital strategy of cord gases and trigger tool (TT) support appropriate clinical decisions regarding TH?*	• Cord or 1 h blood gas pH <7 OR BE≤ −12 • 10 min Apgar ≤5 • assisted ventilation ≥ 10 min from birth n=580 (0.9%) after exclusions (stillborn, died before admission, major congenital anomalies)	Modified (6-point) Sarnat scores hourly for up to 6h; n=60 screened	✓ n=22702 (35.4%)	N=60+16 identified before TT applied Missed = 0	All but 3 infants with probable moderate or severe HIE received TH – these 3 did not meet any eligibility criteria for screening
**Peebles 2024 [21] USA** Hospital of Univ. Pennsylvania (HUP) QI project with defined PDSA cycles	Neonates >35/40 & BW >1800g; October 4 2020 to May 13 2023; N= "~4200 annual births"; *To increase the presence of complete documentation regarding an HIE TH evaluation among infants at risk for HIE from a baseline of 47% to 90% by Jan 2023*	<u>Option 1</u> Cord blood gas pH ≤ 7 or BE≤ −15 <u>Option 2</u> Cord blood pH ≤ 7.15 or BE −10 to −14.9 OR no cord blood gas result AND CPAP/PPV/intubated after birth & continued for ≥10 min, OR 10-min Apgar ≤ 5 and acute perinatal event n=894	6-point neurological exam (CHOP HIE pathway for evaluation of infants who met cord gas criteria – not shown but is quoted in references) n=254	"Cord gases are routinely sent after all deliveries at HUP" *# not reported (?100%)*	*# identified cases: not reported # missed cases: not reported*	The average % of at-risk infants who had an HIE TH evaluation documented in their medical chart increased from 47 to 82%.
**Blecharczyk 2022 [14] USA** Stanford/ Lucille Packard Before and	Infants ≥36 wks, abnormal cord gases (pH 7.0 or base deficit > 10); Aug 2016 to June 2019 vs Jan 2016 to June 2016;	<u>Option 1</u> Cord gases pH< 7.0 or BD>16 mmol/L <u>Option 2</u>	Repeat gases, modified Sarnat (unclear when or how frequently performed)	"Discretion of obstetrics" (usually for concerns	Second epoch 20 cases correctly identified	No change in TH under-utilization during their QI period (but number not provided): Jan 2013

after audit of standardized clinical care pathway	N=11,981 births *To evaluate adoption of standardized clinical care pathway and its impact on clinical care processes with regards to timely evaluation of neonates with umbilical-cord acidemia at risk of HIE.*	pH 7.01 and 7.15 or BD between 10 and 15.9 plus clinical criteria (e.g., perinatal sentinel event" and either low 10 min Apgar or assisted ventilation at birth >10 mins) if milder acidosis n=203 abnormal cord gases in 2nd epoch n=104 in 1st epoch	*# not reported*	about fetal status before or during delivery) *# not reported*	*# missed cases not reported*	to July 2017) Admissions = 734 neonatal encephalopathy or HIE = 104 Epoch 2: (Aug 2017 to Oct 2019) *# admissions = not reported* HIE = 79 TH = 63 (32 of whom didn't meet criteria for gestation, severity of encephalopathy or time of commencement)
**Carlton 2021 [15] USA** Hospital of Univ Wisconsin QI study with retrospective (before) and prospective (after) cohorts	infants admitted to a level IV NICU ≥36 weeks, 1,800g and <6h age *Eligible liveborn population not reported. To determine utilization drift of TH Aim: to increase the number of appropriate TH cases between each misuse TH case*	<u>Option1</u> pH <7.0 or BD >-16mmol/L <u>Option 2</u> pH 7.01 - 7.15 & BD= 10 to 15.9 in conjunction with sentinel event & Apgar of 5 @ 10 mins or assisted ventilation until 10 mins of life *# screened positive not stated*	modified Sarnat score moderate or severe HIE n=79 in second epoch	✕ *# not reported*	Second epoch 20 cases correctly identified, *# missed cases not reported*	No change in TH under-utilization during their QI period (but *no #provided*) Epoch 1: Jan 2013 to July 2017) Admissions = 734 neonatal encephalopathy or HIE = 104 Epoch 2: (Aug 2017 to Oct 2019) *# admissions = not stated* HIE = 79, TH = 63 (32 of whom didn't meet criteria for gestation, severity or time of commencement)
**Mohammed 2022 [20] Canada** Univ. review Calgary Retrospective cohort study	GA >35/40 & <6 hrs of life who were admitted with diagnosis of moderate or severe HIE *Eligible liveborn population not reported To "Evaluate the impact of an outreach education program"*	PPV at 10 min OR cord/postnatal gases pH <7 or BE < -16 *# screened positive not stated*	Modified Sarnat Apgar <5 at 10 min OR PPV at 10 min OR cord/postnatal gases pH <7 or BE < -16 *# screened positive not stated*	✕ *# not reported*	165 infants inborn and outborn had HIE *# missed not reported*	Significant reduction in combined primary outcome of death and/or severe brain injury.
**Takenouchi 2010 [22] USA** New York-Presbyterian	GA > 36/40 with mod to sev HIE who met criteria for head cooling according to CoolCap study criteria	Criteria not reported *# screened positive not stated*	Modified Sarnat scoring and aEEG evidence based on CoolCap study	✕ *# not reported*	45 cases detected *# missed not reported*	Association of death and /or abnormal neurodevelopment with

Retrospective chart review	May 2007 to May 2009<br>*Eligible liveborn population not reported*<br>*Can we predict which infants are at high risk for poor outcomes earlier?*		*# screened positive not stated*			an early postnatal pH <7.00
**Keiffer 2023 [19]**<br>**USA**<br>70 bed level IV NICU<br>Retrospective cohort study	≥36 weeks, 1,800g and <6h age<br>Aug 2019 to Mar 2022<br>*Eligible liveborn population not reported*<br>*To determine long term sustainability of QI methods to decrease TH misuse*	Option 1<br>pH <7.0 or BD >16mmol/L<br>Option 2<br>pH 7.01 to 7.15 AND BD 10 to 15.9 in conjunction with sentinel event AND Apgar of 5 at ten min or assisted ventilation until 10 mins of age<br>*# screened positive not stated*	Modified Sarnat score moderate or severe<br>*# screened positive not stated*	✗<br>*# not reported*	64 patients with HIE; 50 received TH<br>*# missed not reported*	17 cases (34%) "misused" TH Documentation improved to 78%. Lack of documentation was significantly associated with TH guideline misuse
**Beck 2023 [13]**<br>**France** 68 level III NICUs<br>Prospective cohort study	Infants eligible for TH ≥34 weeks from liveborn population N=1,157,846<br>*"To determine clinical and healthcare organisation factors associated with delayed TH"*	Acute perinatal event, abrupt change in FHR (or persistently abnormal), 10 min Apgar <5 or assisted ventilation for 10 mins from birth<br>*# screened positive not stated*	Modified Sarnat, pH <7.15, base deficit >10<br>*# screened positive not stated*	✗<br>*# not reported*	*# screened positive not reported*<br>*# missed not reported*	79.7% achieved TH within 6 hours<br>Delay between birth and call for transfer, undesirable event in transport and decreased annual birth rate associated with ↑ risk of delay
**Khurshid 2011 [18]**<br>**Canada**<br>Hospital for Sick Children<br>Retrospective review	Neonates ≥36 weeks with HIE referred for retrieval by nontertiary centres<br>*Dates – not stated*<br>*Eligible liveborn population not reported*<br>*"To determine if eligible infants are appropriately offered TH and what barriers if any"*	10 min Apgar <5; assisted ventilation for 10 mins from birth; pH <7.0 or BD ≥16<br>*# screened positive not stated*	Mod or sev HIE using modified Sarnat score OR clinical seizures<br>*# screened positive not stated*	✗<br>*# not reported*	41 infants retrieved with HIE evaluated for TH of whom 24 (67%) received TH<br>*# missed not stated*	Consensus reached about inclusion criteria for TH as per screening in this paper. Insufficient timely and accurate identification of eligibility for TH were main reasons for delay in TH
**Goswami 2021 [17]**<br>**Canada**<br>'Quaternary NICU'<br>4x PDSA cycles	Infants undergoing TH<br>*Dates – not stated*<br>*Eligible liveborn population not reported*<br>*Aimed to increase compliance with and quality of cerebral function*	*Criteria not stated*<br>*# screened positive not stated*	*Criteria not stated*<br>*# screened positive not stated*	✗<br>*# not reported*	*# detected not stated*<br>*# missed not stated*	

	monitoring documentation in EMR					
**Chalak 2024 [16] USA** Parkland Health, Dallas County public hospital Prospective cohort study	2012-2023 *Eligible liveborn population not reported* Short report format; aims for study not explicitly stated	*Criteria not explicitly stated* *# screened positive not stated*	"detailed Sarnat scoring by certified physicians" *# screened positive not stated*	✗ *# not reported*	418 infants with any stage of HIE (187 received TH) *# missed not stated*	"Strengths of this study include detailed neurological examination, universal

List of abbreviations: *aEEG= amplitude integrated electroencephalography; BD= base deficit; BE= base excess; BW= birth weight; CPAP= continuous positive airway pressure; EMR= electronic medical record; FHR= fetal heart rate; GA= gestational age; HIE= hypoxic ischaemic encephalopathy; NICU= neonatal intensive care unit; PDSA= Plan-Do-Study-Act ; PPV= positive pressure ventilation; QI= quality improvement; TH= therapeutic hypothermia; TT= MaterMothers Hospital's HIE Trigger Tool*

Supplementary Section S2

List of criteria for cord blood gases at the Mater Mothers' Hospital

a. Any cardiotocograph classified as 'RED' (likely probability of fetal compromise)
b. Meconium staining of amniotic fluid
c. Preterm delivery less than 37 weeks gestation
d. Intrapartum fetal scalp lactate performed
e. Vaginal breech delivery
f. Twin vaginal birth
g. Emergency caesarean section
h. Elective caesarean section if: i. delay or difficulty delivering baby ii. fetal growth restriction iii. pre-eclampsia
i. Prolonged second stage (greater than 3 hours in total)
j. Any instrumental delivery/ operative vaginal birth
k. Shoulder dystocia
l. Intrapartum fever (more than 38 degrees Celsius)
m. Cutting and clamping the cord prior to delivery
n. Flat baby at birth: I. Low Apgar score – less than five at one minute II. Neonatal respiratory depression with requirement for resuscitation
o. Any other situation where Paediatrician attendance is indicated (excluding low risk elective CS).

Cord blood sampling at birth and histopathological examination of the placenta – statewide procedure. Rev. No: 3.02. Document Num: MPPL-04492. Approval: Director Obstetrics & Gynaecology. Released: 21/12/2022. Next review: 21/12/2025.

Supplementary Section S3

Mater Mothers Hospital HIE Trigger Tool.

F3183

Mater Misericordiae Ltd

HYPOXIC ISCHEMIC ENCEPHALOPATHY (HIE) TRIGGER TOOL

Unit Record No. ______________________

Surname ______________________

Given Names ______________________

DOB ______________ Sex ______________

AFFIX PATIENT IDENTIFICATION LABEL HERE

Binding margin - do not write. Do not reproduce by photocopying.
All clinical form creation and amendments must be conducted through Health Informatics.

Infant 35^{+0} weeks gestation with

- ☐ pH<7.00 **or** Base excess worse than or equal to -12 (cord blood or in first hour) ➔ pH .... BE .... **or**
- ☐ Continued need for resuscitation at 10 minutes ➔ ☐ Yes ☐ No **or**
- ☐ 10 minute Apgar score 5 ➔ Apgar .... /10

⬇

Admit to ICN1 or 2 or SCN

(level depending on usual criteria for admission to these levels of care)

⬇

- Capillary, venous or arterial blood gas within the first hour
- Other investigations and cares as warranted by history and clinical condition
- Hourly assessment (by nurse or medical of ficer) using Simplified Sarnat Criteria (below)

⬇

Simplified Sarnat Criteria (assess as many signs as possible)

N = Normal	MILD = Mild	MOD = Moderate	S = Severe	Record actual time of exam 1h	2h	3h	4h	5h	6h
**Severity**	Mild encephalopathy	Moderate encephalopathy	Severe encephalopathy						
**Level of consciousness**	Hyperalert	Decreased - reduced response to non-painful stimulation ("lethargic")	Absent - only responds to painful stimuli ("stupor"); or No or minimal response to pain ("coma")						
**Spontaneous activity**	Normal or increased	Decreased	None						
**Tone***	Normal or increased in trunk AND extremities	Hypotonia - reduced trunk OR extremity tone OR both	Flaccid - no tone						
**Suck reflex**	Normal or incomplete	Incomplete	Absent						
**Moro reflex**	Strong, low threshold	Incomplete	Absent						
**Respiratory abnormality**		Periodic breathing	Apnoea						

*Assess tone in both limbs and trunk/ neck. Presence of hypotonia in either meets the criterion.

⬇

If, at any time point in the first 6 hours, infant meets any:

» **3 criteria in moderate or severe category or both or**
» **2 criteria plus clinical or electrographic seizures**

- Medical officer to document the time and level of encephalopathy in the health record. If equal number of "MOD" or "S" items, level of consciousness decides severity of encephalopathy.
- Refer to neonatal fellow who will assess the baby and discuss initiating therapeutic hypothermia with consultant.

⬇

Infant does not meet criteria by 6 hours of age ➔ Can be considered for routine care on postnatal ward or in NCCU depending on other clinical criteria.

Clinician name:	Designation:	Signature:	Date:

06/18
Ver. 3.00
F3183

HIE TRIGGER TOOL 800

© Copyright Mater Misericordiae Limited. All rights reserved.
